# Diabetes Alters Intracellular Calcium Transients in Cardiac Endothelial Cells

**DOI:** 10.1371/journal.pone.0036840

**Published:** 2012-05-09

**Authors:** Abdul Q. Sheikh, Jennifer R. Hurley, Wei Huang, Toloo Taghian, Andrei Kogan, Hongkwan Cho, Yigang Wang, Daria A. Narmoneva

**Affiliations:** 1 School of Energy, Environmental, Biological and Medical Engineering, University of Cincinnati, Cincinnati, Ohio, United States of America; 2 Department of Pathology and Laboratory Medicine, University of Cincinnati Medical Center, Cincinnati, Ohio, United States of America; 3 Physics Department, University of Cincinnati, Cincinnati, Ohio, United States of America; University of Padova, Italy

## Abstract

Diabetic cardiomyopathy (DCM) is a diabetic complication, which results in myocardial dysfunction independent of other etiological factors. Abnormal intracellular calcium ([Ca^2+^]_i_) homeostasis has been implicated in DCM and may precede clinical manifestation. Studies in cardiomyocytes have shown that diabetes results in impaired [Ca^2+^]_i_ homeostasis due to altered sarcoplasmic reticulum Ca^2+^ ATPase (SERCA) and sodium-calcium exchanger (NCX) activity. Importantly, altered calcium homeostasis may also be involved in diabetes-associated endothelial dysfunction, including impaired endothelium-dependent relaxation and a diminished capacity to generate nitric oxide (NO), elevated cell adhesion molecules, and decreased angiogenic growth factors. However, the effect of diabetes on Ca^2+^ regulatory mechanisms in cardiac endothelial cells (CECs) remains unknown. The objective of this study was to determine the effect of diabetes on [Ca^2+^]_i_ homeostasis in CECs in the rat model (streptozotocin-induced) of DCM. DCM-associated cardiac fibrosis was confirmed using picrosirius red staining of the myocardium. CECs isolated from the myocardium of diabetic and wild-type rats were loaded with Fura-2, and UTP-evoked [Ca^2+^]_i_ transients were compared under various combinations of SERCA, sarcoplasmic reticulum Ca^2+^ ATPase (PMCA) and NCX inhibitors. Diabetes resulted in significant alterations in SERCA and NCX activities in CECs during [Ca^2+^]_i_ sequestration and efflux, respectively, while no difference in PMCA activity between diabetic and wild-type cells was observed. These results improve our understanding of how diabetes affects calcium regulation in CECs, and may contribute to the development of new therapies for DCM treatment.

## Introduction

Diabetes mellitus is a growing epidemic in the United States that afflicts 8.3% of the total US population [1]. Diabetic cardiomyopathy (DCM) is a diabetes-associated condition, which is defined as changes in the structure and function of the myocardium, independent of other etiological factors [2]. Despite the potential importance of DCM, the complex and multifactorial nature of the cellular and molecular perturbations, which may alter myocardial function, remain poorly understood.

Studies in animal models have shown that diabetes results in abnormal calcium (Ca^2+^) homeostasis that may precede clinically manifested DCM-related cardiac dysfunction [3], [4]. Importantly, alterations in Ca^2+^ regulatory mechanisms are a hallmark of cardiomyopathy and heart failure in human patients [4]. During normal myocardial contraction, intracellular Ca^2+^ concentration ([Ca^2+^]_i_) in cardiomyocytes increases due to Ca^2+^ entry via Ca^2+^ channels and inositol trisphosphate (IP3) mediated Ca^2+^ release from sarco/endoplasmic reticulum (SER). Cardiac relaxation is subsequently initiated by the Ca^2+^ decay process, which is governed by Ca^2+^ sequestration and Ca^2+^ efflux mechanisms. During Ca^2+^ sequestration, the sarcoplasmic reticulum Ca^2+^ ATPase pumps (SERCA) re-sequester the Ca^2+^ from the cytosol back to the SER. Similarly the Ca^2+^ efflux mechanism extrudes Ca^2+^ via plasma membrane Ca^2+^ ATPase pumps (PMCA), and sodium- calcium exchangers (NCX) [5]–[7].

In recent reports, diabetic cardiomyocytes displayed reduced SERCA activity and Ca^2+^ sequestration, as well as impaired NCX function [8], [9]. Similarly, aortic and arterial smooth muscle cells isolated from diabetic rats have shown reduced Ca^2+^ signaling [10] and abnormalities in SER Ca^2+^ mobilization [8]. Furthermore, SERCA overexpression in transgenic mice resulted in the protection against cardiac dysfunction in streptozotocin-induced diabetes [11]. Overall, these results suggest potential mechanisms for diabetes-induced disruptions in Ca^2+^ homeostasis.

It is well established that endothelium plays an essential role in vascular function, including nitric oxide (NO) -mediated modulation of vascular tone, control of adhesion between the vessel and cells circulating in the blood, and regulation of local cell and vessel growth via a variety of growth factors. Calcium is an important regulator of endothelial function that can mediate each of these pathways, including signaling via NO [12], [13], cell adhesion molecules [14], [15], and receptor tyrosine kinases [16], [17]. Importantly, diabetes is associated with impaired endothelium-dependent relaxation and diminished capacity to generate NO [18]–[21], elevated cell adhesion molecules [22], [23], and decreased angiogenic growth factors [24], [25]. Despite the available evidence of a link between calcium signaling and endothelial cell function, as well as documented endothelial dysfunction in diabetes, the possible effects of diabetes on Ca^2+^ signaling in endothelial cells are not known, which may delay the development of vascular therapies for diabetic patients. In particular, the information regarding the effects of diabetes on Ca^2+^ regulation in cardiac endothelium is especially important, because cardiac endothelial cells (CECs) are instrumental in normal myocardial function, including endocardium interactions, myocardial capillaries, vascular permeability and cardiac tissue remodeling. Therefore, this information is essential for a better understanding of mechanisms for the impaired cardiac function in DCM and for the development of new therapies to treat this condition.

**Figure 1:**
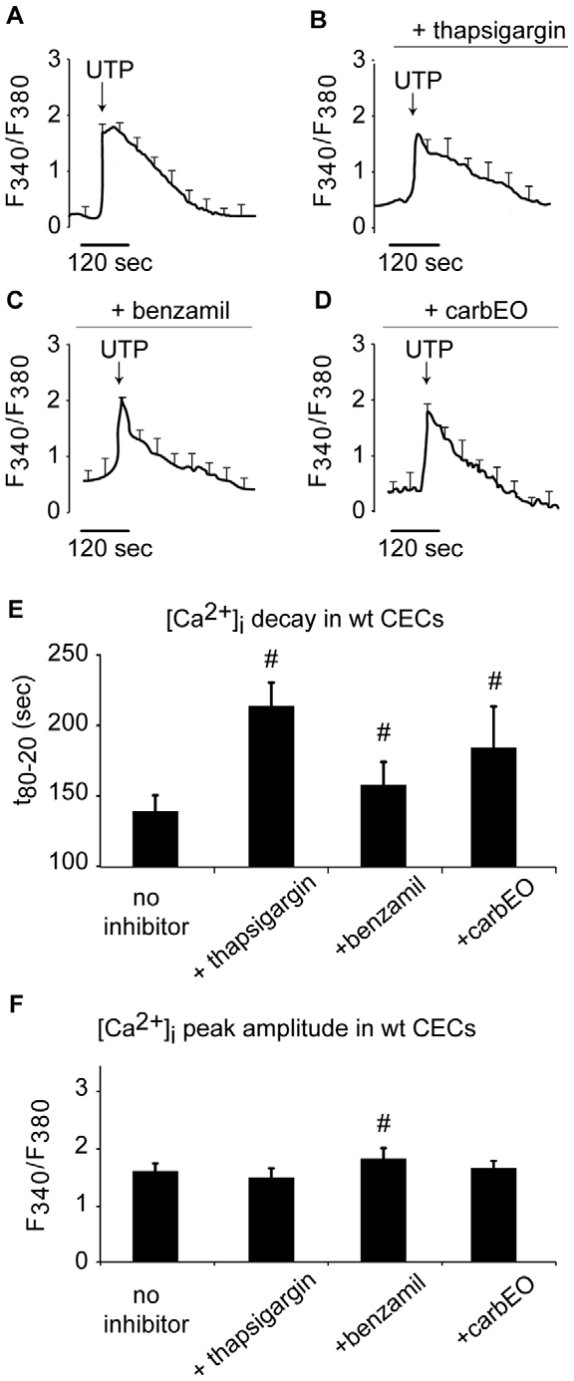
Effect of SERCA, NCX and PMCA inhibition on [Ca^2+^]_i_ transients in wild-type CECs. [Ca^2+^]_i_ transients were evoked by UTP in the absence (A) or presence of SERCA (thapsigargin), NCX (benzamil) and PMCA (carboxyeosin (carbEO)) inhibitors in wild-type CECs (B, C, and D). Successful inhibition of SERCA, NCX and PMCA in wild-type CECs was demonstrated by an increased [Ca^2+^]_i_ decay time (E; from 80% to 20% of its peak amplitude) in the presence of inhibitors (Ca^2+^ free medium). [Ca^2+^]_i_ peak amplitude in the presence of NCX inhibitor was significantly greater then no inhibitor controls (F), while SERCA and PMCA inhibitors had no affect on peak amplitude (#: p<0.05; cell no.: A: 79, B: 88; C: 85, D: 92).

**Figure 2:**
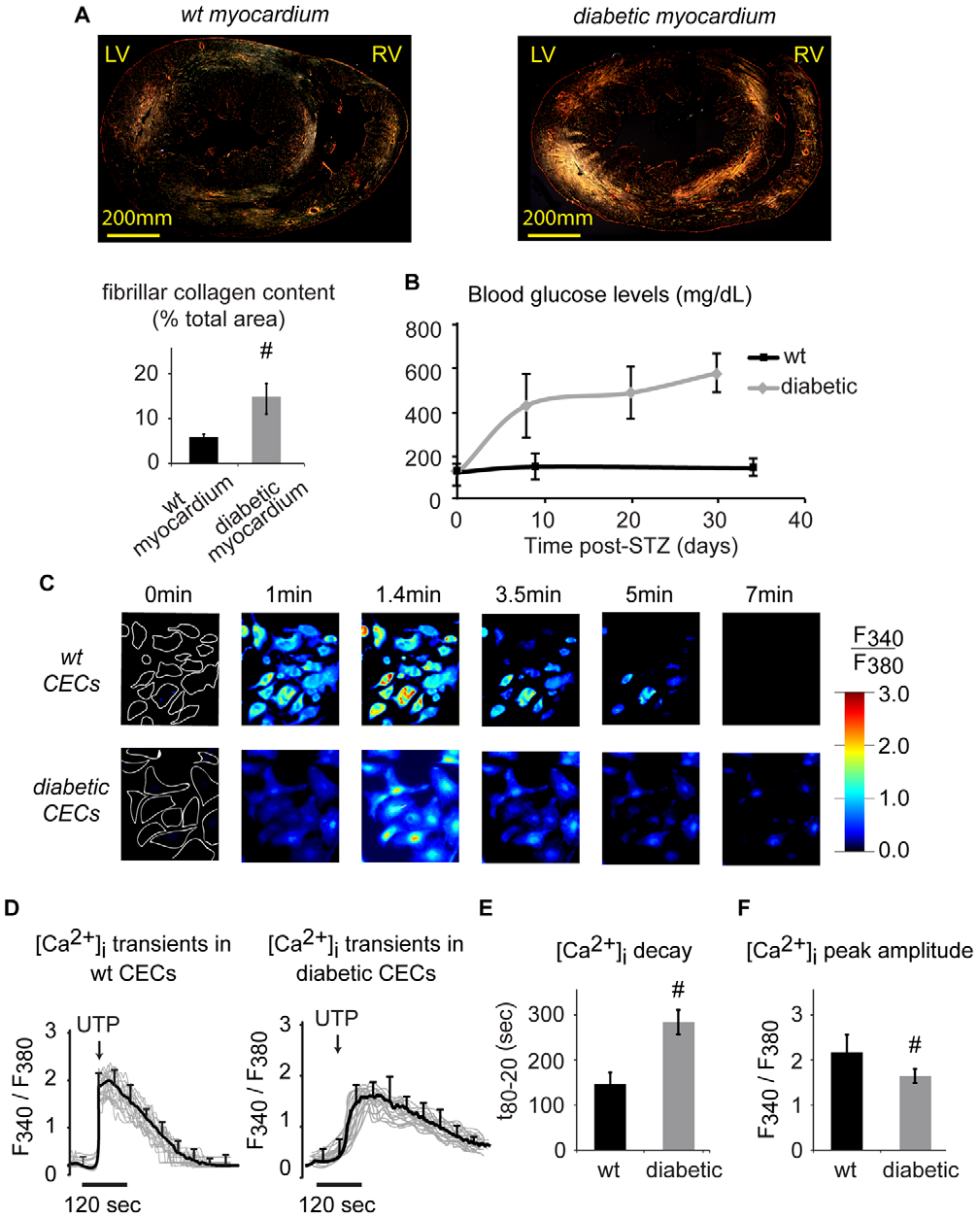
Diabetes induces cardiac fibrosis and alters [Ca^2+^]_i_ transients in CECs. (A): Analysis of the Picrosirius red staining of diabetic myocardium using polarized light microscopy showed the presence of cardiac fibrosis with significantly increased area of fibrillar collagen fibers (yellow, orange or red, middle panel), as compared to mostly reticular fibers (green, left panel) in the myocardium of age-matched wild-type animals (LV, RV: left or right ventricle). (B): Rats injected with STZ showed significant and rapid hyperglycemia by day 5 that persisted throughout the study. (C–F): Significant differences in UTP-evoked [Ca^2+^]_i_ transients were observed between wild-type and diabetic CECs (C, D). [Ca^2+^]_i_ transients in individual cells (outlined in white in the first frame) obtained using ratiometric analysis are shown as grey lines, with the average transient as a black line. Analysis of transients (E–F) demonstrated significantly longer [Ca^2+^]_i_ decay time and smaller peak amplitude in diabetic CECs, as compared to wild-type CECs (#: p<0.05; cell no: 206 (wt), 195 (db)).

**Figure 3:**
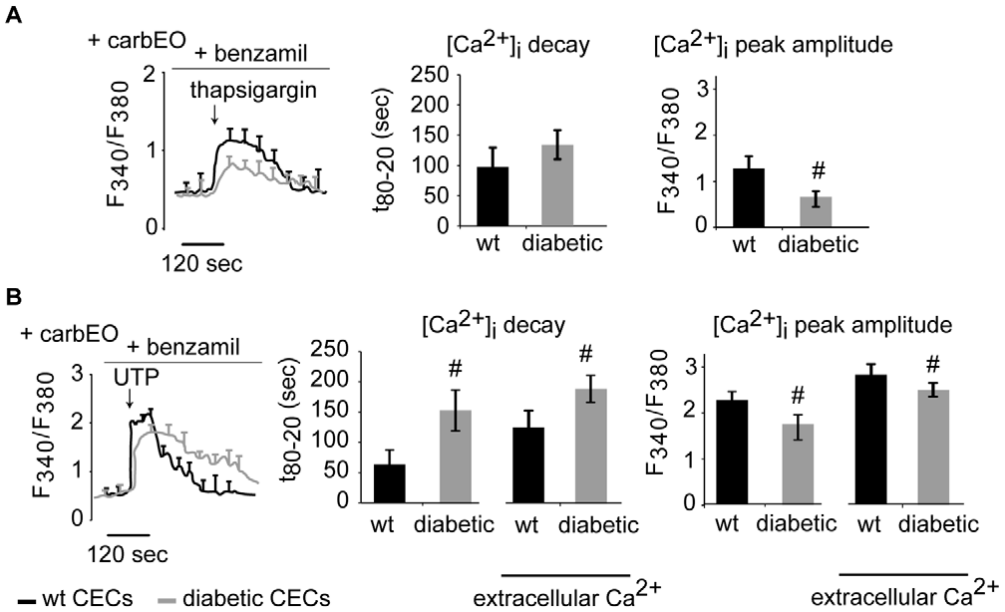
Diabetes alters SERCA-mediated Ca^2+^ sequestration. Cells were treated with PMCA (carbEO) and NCX (benzamil) inhibitors to study SERCA activity during Ca^2+^ re-sequestration without the contribution of Ca^2+^ efflux mechanism. (A): Slow and long-lasting Ca^2+^ transients were evoked by thapsigargin (in Ca^2+^ free medium), with no significant difference in decay time between wild-type and diabetic CECs. However the peak amplitude in diabetic CEC was significantly smaller then in wild-type group. (B): UTP-evoked [Ca^2+^]_i_ transients in diabetic cells had significantly longer decay time and smaller peak amplitude in the absence or presence of extracellular Ca^2+^ (#: p<0.05; cell no: A: 82 (wt), 76 (db); B: 73 (wt), 78 (db)).

**Figure 4:**
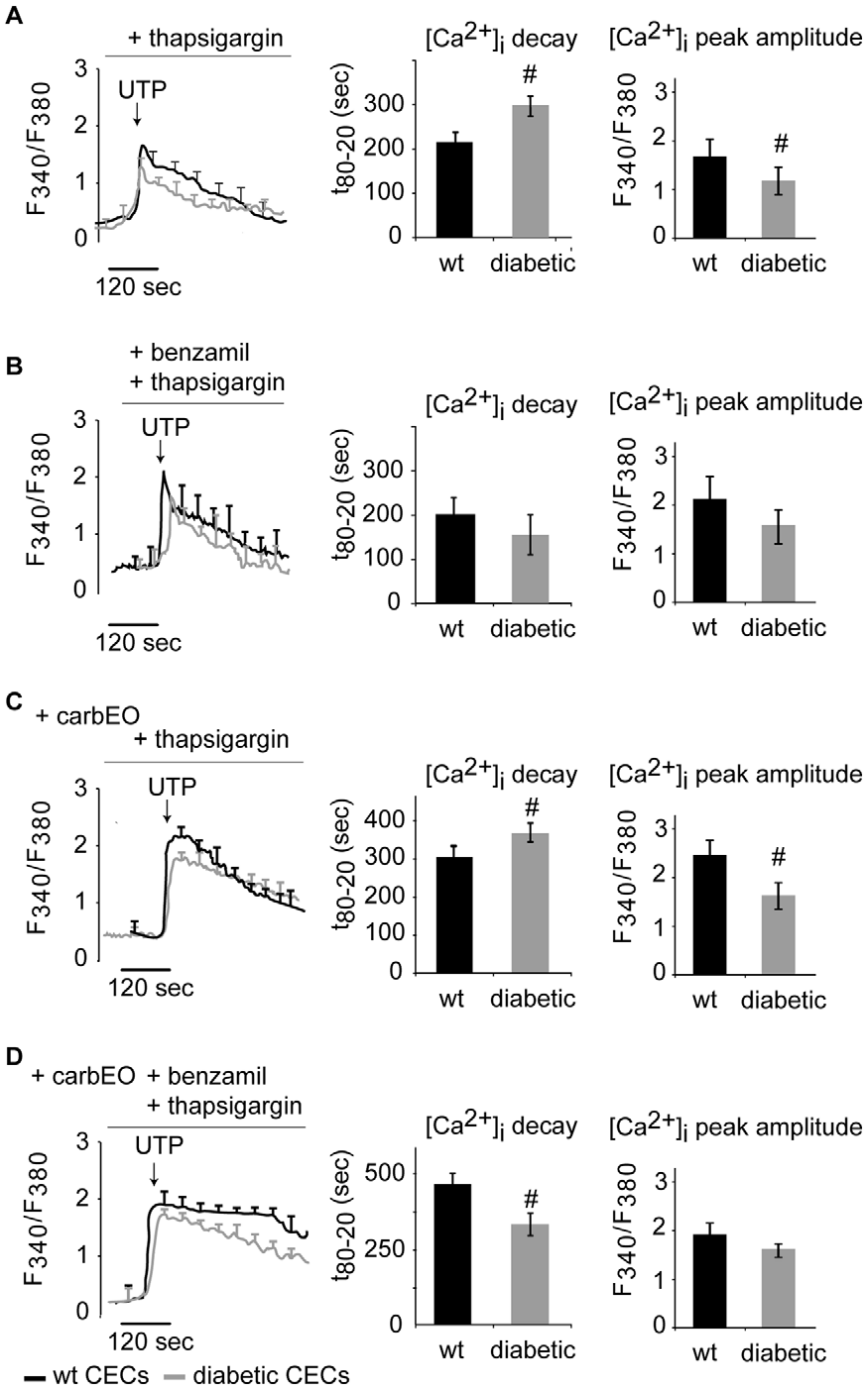
Diabetes alters Ca^2+^ NCX activity without affecting PMCA-mediated efflux. (A): CECs were treated with SERCA inhibitor (thapsigargin) to study the effect of diabetes on intracellular Ca^2+^ efflux mechanism without contributions from Ca^2+^ sequestration mechanism. UTP-evoked [Ca^2+^]_i_ decay time was significantly increased and the peak amplitude was decreased in diabetic CECs, as compared to wild-type controls. (B): CECs were treated with SERCA (thapsigargin) and NCX (benzamil) inhibitors to study the effect of diabetes on PMCA-mediated Ca^2+^ efflux. Under these conditions, there was no significant difference in UTP-evoked [Ca^2+^]_i_ transients between diabetic and wild-type CECs. (C): CECs were treated with SERCA (thapsigargin) and PMCA (carbEO) inhibitors to study the effect of diabetes on NCX-mediated Ca^2+^ efflux. In diabetic CECs, NCX activity was significantly reduced as indicated by increased decay time of UTP-evoked [Ca^2+^]_i_ transients. The peak amplitude was also significantly reduced in diabetic CECs under these conditions. (D): To confirm effect of diabetes on [Ca^2+^]_i_ transients, Ca^2+^ decay was inhibited by treating CECs with SERCA (thapsigargin), PMCA (carbEO) and NCX (benzamil) inhibitors. In wild-type CECs, [Ca^2+^]_i_ decay was completely blocked, while diabetic CECs still showed Ca^2+^ removal from the cytosol, with [Ca^2+^]_i_ decay time significantly faster than that in wild-type CECs. No change in peak amplitude was observed under these conditions (#: p<0.05; cell no. A: 69 (wt), 93 (db); B: 83 (wt), 73(db); C: 74 (wt), 71 (db); D 76 (wt), 72 (db)).

**Figure 5:**
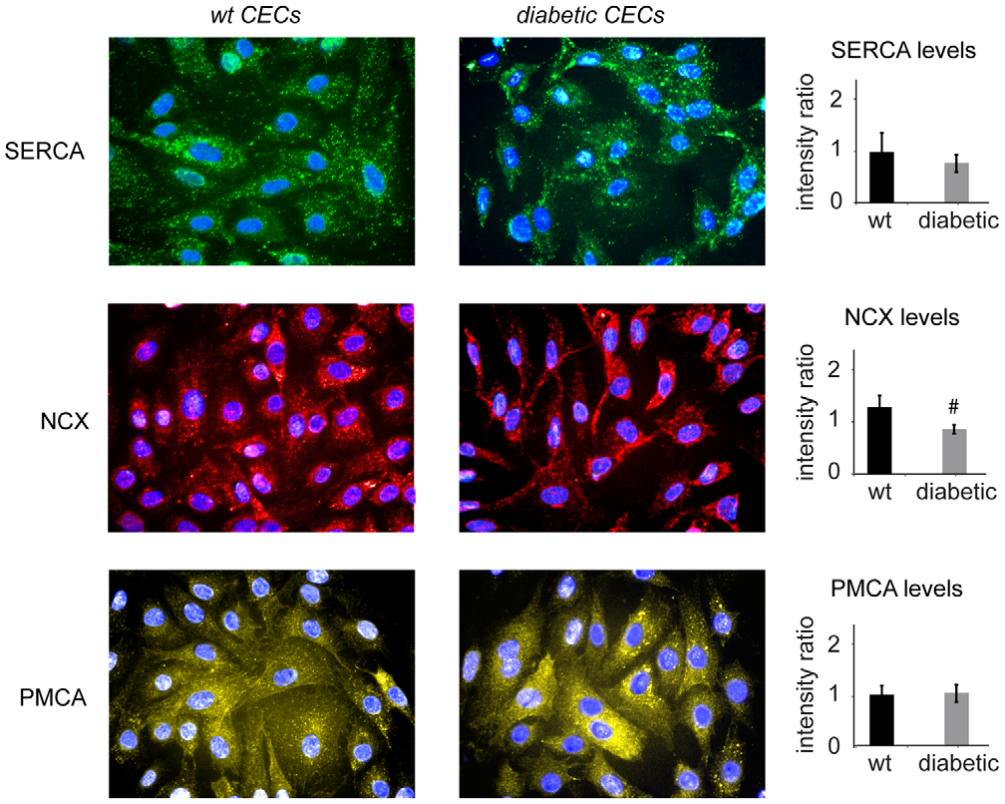
Diabetes results in decreased NCX protein expression, with no difference in SERCA and PMCA expression levels. Semi-quantitative analysis of immunofluorescent staining of CECs showed that diabetes significantly reduces the expression levels of NCX protein as compared to wild-type (NCX: red; blue: cell nuclei, #: p<0.05). No significant difference in the expression levels of SERCA and PMCA was observed in diabetic cells as compared to wild-type (SERCA: green; PMCA: yellow; blue: cell nuclei).

The objective of this study was to determine the effect of diabetes on intracellular Ca^2+^ homeostasis in CECs in a rat model (streptozotocin-induced) of DCM. Uridine-5′-triphosphate (UTP) -evoked [Ca^2+^]_i_ transients were compared between diabetic and wild-type CECs under various combinations of SERCA, PMCA and NCX inhibitors. Our results demonstrate that diabetes results in altered Ca^2+^ homeostasis in endothelial cells via decreases in SERCA and NCX activities during Ca^2+^ sequestration and Ca^2+^ efflux, respectively.

## Results

### Effect of SERCA, NCX and PMCA inhibitors on [Ca^2+^]_i_ transients in wild-type CECs

The objective of these experiments was to establish the baseline for normal (wild-type) CEC response to SERCA, NCX and PMCA inhibitors. Consistent with previous studies [26], UTP (a P_2Y2_ receptor agonist) evoked a [Ca^2+^]_i_ transient which decayed to basal level (Fig. 1A). The decay time of UTP-evoked [Ca^2+^]_i_ transient was significantly higher in the presence of SERCA inhibitor, thapsigargin (Fig. 1B, p<0.05). The effect of thapsigargin was further confirmed by the subsequent addition of 2.0 mmol/L Ca^2+^ to the medium, which evoked a marked [Ca^2+^]_i_ increase due to the depletion of SER and Ca^2+^ influx through store-operated Ca^2+^ channels (data not shown).

As expected, in the presence of NCX inhibitor (benzamil), the Ca^2+^ decay time (Fig. 1C) was significantly increased in CECs as compared to no inhibitor controls (Fig. 1E, p<0.05). Similarly, the presence of carboxyeosin (carbEO), a selective PMCA inhibitor resulted in a significant increase in the decay time of UTP-evoked [Ca^2+^]_i_ transient, as compared to no inhibitor treated CECs (Fig. 1D, E, p<0.05). These experiments were performed in Ca^2+^ free solution to avoid any contributions of Ca^2+^ influx from the extracellular space. To further validate these results, experiments were repeated in the presence of extracellular Ca^2+^, which also resulted in slower [Ca^2+^]_i_ decay time in wild-type CECs (data not shown) consistent with previous reports [27].

The [Ca^2+^]_i_ peak amplitude in wild-type CECs remained similar in the presence of SERCA and PMCA inhibitors as compared to no inhibitor controls. However, cells treated with NCX inhibitor showed a higher [Ca^2+^]_i_ peak as compared to no inhibitor controls (Fig. 1F, p<0.05).

### Cardiac fibrosis and increase in blood glucose levels after STZ injection

Measurement of blood glucose levels after STZ injection showed significant and rapid hyperglycemia by day 5, which persisted until the end of the experiments (Fig. 2B). The blood glucose levels of the wild-type control animals remained in the physiological range for the duration of the study. Picrosirius red staining of the rat myocardium under polarized light showed significantly higher levels of cardiac fibrosis (yellow, orange, or red) in diabetic animals, as compared to tissue from age- and strain-matched wild-type control animals (p<0.05) [28], where mostly reticular, non-fibrillar collagen (green) was present (Fig. 2A). Interestingly, the values for the fibrillar collagen content in both groups are ∼2-fold higher than those reported in the literature [28], [29]. This may be due to the differences in methodology used to calculate collagen area fraction, including magnification (2X in this study vs. 200X in previous reports), the analysis of the sections of the whole heart vs. left ventricle only, and the normalization procedure (i.e. using the area obtained by tracing the myocardium to exclude voids in this study vs. the ratio of stained area to the unstained area in previous reports [28], [29]). Importantly, despite the differences in the absolute values, the relative increase (approximately 2.5-fold) in the fibrillar collagen content of the diabetic hearts as compared to the normal tissue is consistent with previous findings [28], [29].

### [Ca^2+^]_i_ transients are disrupted in diabetic CECs

In wild-type CECs, UTP evoked a [Ca^2+^]_i_ transient, which gradually decayed with time (Fig. 2C, D). Interestingly, in diabetic CECs, the [Ca^2+^]_i_ transient decay was markedly delayed (Fig. 2C, D). Ratiometric analysis showed that in diabetic CECs, the decay time was significantly longer (Fig. 2E, p<0.05), and the [Ca^2+^]_i_ peak amplitude was significantly smaller, as compared to wild-type CECs (Fig. 2F, p<0.05).

### Altered Ca^2+^ re-sequestration contributes to changes in [Ca^2+^]_i_ transients in diabetic CECs

[Ca^2+^]_i_ transients were first evoked by thapsigargin to explicitly examine Ca^2+^ depletion from SER in a Ca^2+^ free medium [30]. In diabetic cells, the peak amplitude was significantly smaller (db: 0.519±0.12 vs. wt: 0.723±0.05, cell no: 31 (db), 42(wt), p<0.05), and decay time was significantly longer (db: 131±15 vs. wt: 116±9, cell no: 42 (db), 38 (wt), p<0.05), as compared to wild-type CECs, indicating possible alterations in SERCA mediated Ca^2+^ responses in diabetic CECs.

To further examine SERCA-mediated Ca^2+^ sequestration without the contribution of Ca^2+^ efflux mechanism during [Ca^2+^]_i_ decay, CECs were treated with PMCA (carboxyeosin) and NCX (benzamil) inhibitors. Under these conditions, thapsigargin induced a slow and long-lasting Ca^2+^ transient in wild-type CECs. Diabetic CECs showed a similar response, with [Ca^2+^]_i_ decay time similar to that of wild-type CECs (Fig. 3A), while [Ca^2+^]_i_ peak amplitude in diabetic CECs was significantly smaller than in wild-type CECs (Fig. 3A, p<0.05). Interestingly, when [Ca^2+^]_i_ transient was evoked using UTP in the presence of PMCA (carboxyeosin) and NCX (benzamil) inhibitors, the [Ca^2+^]_i_ decay time in diabetic CECs was significantly increased and the peak amplitude was significantly decreased as compared to wild-type CECs (Fig. 3B, p<0.05). Results for [Ca^2+^]_i_ transients evoked in the presence of extracellular Ca^2+^ (2 mM CaCl_2_ added) were similar, with significantly increased [Ca^2+^]_i_ decay time and decreased peak amplitude in diabetic CECs as compared to wild-type (Fig. 3B, p<0.05).

### Ca^2+^ efflux mechanism is altered in diabetic CECs

CECs were treated with thapsigargin to block the contribution of the Ca^2+^ sequestration mechanism during [Ca^2+^]_i_ decay. The observed [Ca^2+^]_i_ decay time was significantly increased in diabetic CECs, as compared to wild-type cells (Fig. 4A, p<0.05), indicating impaired Ca^2+^ efflux in diabetic cells. A significant decrease in [Ca^2+^]_i_ peak amplitude was also observed in diabetic CECs (Fig. 4A, p<0.05). The subsequent experiments were performed to determine whether diabetes affects Ca^2+^ efflux mechanism either via altered PMCA or NCX activity, or a combination of both.

### [Ca^2+^]_i_ extrusion by PMCA during Ca^2+^ efflux is not altered in diabetic CECs

To compare PMCA activity in wild-type and diabetic CECs, cells were treated with SERCA and NCX inhibitors (thapsigargin and benzamil, respectively) to exclude the contributions of SERCA-mediated Ca^2+^ sequestration and NCX-mediated Ca^2+^ efflux. Under these conditions, the UTP-evoked [Ca^2+^]_i_ transient did not show a significant difference in [Ca^2+^]_i_ decay time in diabetic CECs, as compared to wild-type (Fig. 4B, p<0.05), indicating that PMCA effectively extrudes Ca^2+^ in both diabetic and wild-type CECs. Furthermore, no differences in [Ca^2+^]_i_ peak amplitudes of diabetic and wild-type CECs were observed under these conditions.

### Altered NCX activity affects Ca^2+^ efflux mechanism in diabetic CECs

[Ca^2+^]_i_ transients were evoked by UTP in the presence of SERCA and PMCA inhibitors (thapsigargin and carboxyeosin, respectively) to exclude contributions of SERCA-mediated Ca^2+^ sequestration and PMCA-mediated Ca^2+^ efflux. Interestingly, the [Ca^2+^]_i_ decay time was significantly increased in diabetic CECs as compared to wild-type (Fig. 4C, p<0.05), indicating altered NCX activity during the Ca^2+^ efflux. [Ca^2+^]_i_ peak amplitude in diabetic CECs was also significantly reduced as compared to wild-type CECs. To further confirm these results, [Ca^2+^]_i_ decay was inhibited by treating CECs with SERCA (thapsigargin), PMCA (carboxyeosin) and NCX (benzamil) inhibitors. Under these conditions, the [Ca^2+^]_i_ decay in wild-type CECs was completely blocked (Fig. 4D). However, in diabetic CECs, there was still Ca^2+^ removal from the cytosol, with [Ca^2+^]_i_ decay time significantly faster than in wild-type CECs (Fig. 4D, p<0.05). Under these conditions, no significant differences in the peak amplitudes of diabetic and wild-type CECs were observed. The reasons for the residual Ca^2+^ extrusion (Fig. 4D) are not clear, and further studies are required to investigate the possible cross talk between SERCA and PMCA pumps.

### Diabetes alters NCX expression levels, but does not effect SERCA and NCX levels

Immunofluorescent staining for SERCA, NCX and PMCA protein expression levels indicated that NCX expression levels in diabetic CECs were significantly reduced as compared to wild-type CECs (Fig. 5, p<0.05), while no effects of diabetes on the expression levels of SERCA and PMCA proteins where observed (Fig. 5).

## Discussion

The results of this study demonstrate that diabetes results in significantly altered [Ca^2+^]_i_ transients via disruptions in SERCA-mediated Ca^2+^ sequestration and NCX-mediated Ca^2+^ efflux mechanisms. To the best of our knowledge, this is the first report of the effect of diabetes on intracellular Ca^2+^ regulatory mechanisms and overall Ca^2+^ homeostasis in the cardiac endothelium. These results are consistent with previous studies in diabetic cardiomyocytes, where altered Ca^2+^ handling was observed in cells isolated from diabetic mice [31] and rats [32]. Similarly, smooth muscle cells isolated from the aortas of type I diabetic rats showed significant alterations in SER-Ca^2+^ kinetics, resulting in the development of diabetic vascular complications [8]. Our results demonstrate that in diabetic CECs, alterations in [Ca^2+^]_i_ transients involve a change in the Ca^2+^ sequestration mechanism. This change in Ca^2+^ sequestration is due to the reduced activity of SERCA to re-sequester Ca^2+^ upon UTP activation. The effect of reduced SERCA activity during Ca^2+^ sequestration in diabetic CECs appeared more prominent during Ca^2+^ decay, which may be due to the fact that SERCA has a higher affinity for Ca^2+^ as compared to PMCA [5], [7], [33]. Our results suggest that the reduced Ca^2+^ sequestration was independent of SERCA protein expression, since staining for SERCA protein remained unchanged in diabetic CECs. Taken together, these results indicate a common mechanism for diabetes-induced alterations in Ca^2+^ sequestration among several cell types, including cardiomyocytes, endothelial cells and smooth muscle cells.

Our results show that NCX activity during Ca^2+^ efflux is altered in diabetic CECs, as compared to wild-type controls. The exact role of the NCX in endothelial cells is still unclear. However, it is generally accepted that this exchanger normally functions (during the resting stage or when [Ca^2+^]_i_ is high) to extrude Ca^2+^ out of the cell [33]. Because of its bidirectional property, it is also thought to play a critical role in Ca^2+^ influx when the intracellular Na^+^ concentration is high enough to drive the NCX. Our findings of a decrease in NCX activity (with no changes in PMCA activity) in diabetic CECs suggest that the ability of diabetic CECs to maintain Ca^2+^ homeostasis is compromised, which may contribute to abnormal cardiac function. This reduced ability of NCX to extrude Ca^2+^ may also be related to the reduced NCX protein levels as shown by the immunofluorescent staining analysis of diabetic CECs. Our results of altered NCX activity and reduced protein expression levels in diabetic CECs are consistent with previous studies in cardiomyocytes [34], [35]. In contrast, Hill et al. [36] showed that in porcine coronary artery smooth muscle cells, the diabetic condition does not affect Ca^2+^ efflux via NCX, while resulting in altered Ca^2+^ efflux via PMCA. Our results also suggest that in diabetic CECs, PMCA effectively extrudes Ca^2+^ in the presence of SERCA and NCX inhibitors.

Our results show that while diabetic conditions result in impaired function of SERCA and NCX, there are no differences in PMCA function and protein expression between diabetic and wild-type cells. These results are consistent with those by Hill et al. [36], where PMCA activity in diabetic smooth muscle cells was similar to that in wild-type cells in the absence of a functional SERCA. Our results also corroborate those of van Breemen et al. [37] in smooth muscle cells, which demonstrated that upon inhibition of NCX and SERCA, Ca^2+^ is primarily extruded by the PMCA. Overall, our findings suggest that abnormal endothelium-dependent relaxation and diminished capacity to generate NO in diabetes [19] may result from altered Ca^2+^ transients due to impaired NCX and SERCA, which can reduce activation of eNOS and NO production by endothelial cells [38]. Also, altered Ca^2+^ transients in diabetic cells may affect ICAM-1-coupled cytoskeletal rearrangements and endothelial cell interactions with inflammatory cells [14], consistent with upregulation of ICAM-1 observed in diabetic patients [22].

In this study, we specifically focused on the effects of DCM on intracellular calcium regulatory mechanisms (calcium sequestration and efflux), and therefore, the experiments were performed in the absence of extracellular calcium (unless otherwise stated). However, in order to elucidate the mechanisms for DCM-induced alterations in overall calcium signaling, information regarding the effects of diabetic conditions on store operated calcium entry is important as well, and will be the subject of future studies.

While the development of DCM and the associated mechanisms are complex and multifactorial, impaired Ca^2+^ homeostasis has been identified as a hallmark abnormality for this disorder [3]. Numerous approaches have been suggested to treat DCM by increasing SERCA and NCX expression [11]. In addition, Ca^2+^ dependent signaling pathways (such as PKC activity [39]) have been identified as possible therapeutic strategies. Information regarding how diabetes affects Ca^2+^ homeostasis in cardiac endothelium is paramount because Ca^2+^ dependent pathways in CECs are involved in endocardium interactions, vascularization and nitric oxide-mediated cardiac hypertrophy. Furthermore, it has been recently shown that endothelial cells may be directly involved in diabetes–induced cardiac fibrosis via endothelial-to-mesenchymal transition [40]. Therefore, the results of this study provide a new insight into the effects of diabetes on Ca^2+^ regulation in CECs, which may contribute to the design of therapeutic approaches to target abnormal Ca^2+^ homeostasis in diabetic cardiomyopathy. Further studies will be required to determine the exact molecular mechanisms for the observed effects.

## Materials and Methods

### Ethics statement

All procedures were performed according to the protocols approved by the University of Cincinnati Institutional Animal Care and Use Committee (IACUC Protocol # 09-06-24-01).

### Animal model of type I diabetes

In this study, a rat model of type I (streptozotocin-induced) diabetes and non-atherogenic diabetic cardiomyopathy [41] was used. Eight-week-old female Sprague Dawley rats (SAS SD Strain 400, Charles River) were injected intraperitoneally with 70 mg/kg streptozotocin (STZ, Sigma-Aldrich) and allowed to develop type I diabetes for six weeks [41], with diabetic phenotype confirmed by serum glucose levels greater than 300 mg/dl. To assess DCM-associated cardiac fibrosis, fibrillar collagen content was measured using picrosirius red staining of paraffin sections of myocardium [42]. The stained sections were observed under polarized light and photographed with the same exposure time for each section. The percentage of fibrillar collagen (yellow, red or orange color) or non-fibrillar collagen (green color) relative to the total area of the myocardium (traced by hand to exclude void areas) was determined using computer-assisted analysis (Adobe Photoshop CS3, Adobe Systems Incorporated, CA).

### Cardiac endothelial cell isolation and culture

Cardiac cells were isolated from the myocardium of diabetic and wild-type rats as described previously [43]. CECs were sorted from cardiac cell mixture using PECAM-1- and ICAM-2-conjugated magnetic beads (Invitrogen Corporation, CA). Endothelial phenotype was confirmed using von Willebrand Factor staining (Sigma-Aldrich, MO), with >95% cells positively stained. CECs were cultured in medium M199 (HyClone, UT) with 10% fetal bovine serum (Atlanta Biologicals, GA), 1% antibiotic/antimycotic (AtlantaBiologicals, GA), 1% heparin (Sigma-Aldrich, MO) and 10 ng/mL endothelial growth factor supplement (Sigma-Aldrich, MO). CECs from passages 4–9 were used for all experiments. Prior to Ca^2+^ experiments, CECs were trypsinized and seeded on multi-well glass coverslips (Sigma-Aldrich, MO) for 24 hours.

### Measurements of [Ca^2+^]_i_ transients

To exclude the contribution of extracellular Ca^2+^ influx during [Ca^2+^]_i_ transient, experiments were performed in a Ca^2+^ free medium (Hanks’ balanced salt solution without Ca^2+^ and Mg^2+^, supplemented with 25 mM HEPES (Invitrogen Corporation, CA), 0.5% bovine serum albumin (Atlanta Biologicals, GA), 2.5 mM probenecid (Sigma-Aldrich, MO), pH 7.45) and 0.5 mM EGTA (calcium buffer), unless otherwise stated. Wild-type and diabetic CECs were loaded with 5 μM Ca^2+^ sensitive indicator Fura-2 AM (Sigma-Aldrich, MO) for 40 minutes at room temperature. In some experiments, extracellular Ca^2+^ was incorporated by adding 2 mM CaCl_2_ in the Ca^2+^ free medium without EGTA. [Ca^2+^]_i_ transients were evoked in CECs by adding 10 µM UTP (Invitrogen Corporation, CA). UTP has been shown to act on P-type purinergic receptors (P_2Y2_), which leads to a G protein-mediated activation of phospholipase C, followed by inositol 1,4,5-trisphosphate (IP3) formation and the release of Ca^2+^ from IP3 sensitive Ca^2+^ stores (mainly from SER) [26]. The imaging setup included an Olympus IX81 inverted fluorescent microscope (Olympus, PA) equipped with cooled charged couple device camera, automated filter wheel (340 nm and 380 nm excitation wavelengths) and a Fura-2 filter cube (400 diachronic and 510 nm wide band emission filter). After UTP administration, images at 340 nm (bound Ca2+) and 380 nm (unbound Ca^2+^) excitation wavelengths were recorded at 510 nm emission after every 250 msec for at least 20 minutes. For each time point, F340/F380 ratiometric analysis was performed by determining the ratio of 340 nm and the corresponding 380 nm image (ImagePro software, Media Cybernetics Inc., MD). Regions of interest outlining cell boundaries on the ratio image were defined, and the variations in fluorescent intensity of selected cells were tabulated [44]. The individual traces (shown in gray, Fig. 2D) were then used to obtain the average intensity as a function of time (shown in black in Fig. 2D). For every treatment group, each experiment was repeated at least 3 times using fresh cells, and at least n = 70 individual cell traces were analyzed to obtain the average intensity plot as shown in Fig. 2D. Standard deviation was calculated at every time point, and the representative SD error bars are shown for every 10^th^ point.

### Inhibitor studies

To assess the activities of SERCA, PMCA and NCX during the decay of [Ca^2+^]_i_ transients, wild-type CECs were treated with 1 μM thapsigargin (Sigma-Aldrich, MO), a selective SERCA inhibitor [45], 100 μM benzamil (Sigma-Aldrich, MO), an NCX inhibitor [46] or 25 μM carboxyeosin ester (carbEO, Sigma-Aldrich, MO), a selective PMCA inhibitor [47] (Fig. 1). Cells were incubated in an inhibitor-containing medium for 30 minutes prior to Fura-2 administration. For Ca^2+^ sequestration mechanism studies (SERCA activity), [Ca^2+^]_i_ transients were first evoked using 1 μM thapsigargin, followed by 10 μM UTP in a Ca^2+^ free medium. During these experiments, the Ca^2+^ efflux was inhibited using PMCA and NCX inhibitors at all times. To examine the Ca^2+^ efflux mechanism (PMCA and NCX activities), diabetic and wild-type CECs were treated with NCX inhibitor (benzamil) or PMCA inhibitor (carboxyeosin). These experiments were performed under the following conditions: a) SERCA activity was inhibited at all times (thapsigargin present in the medium), b) NCX activity was inhibited to examine PMCA activity, and c) PMCA activity was inhibited to examine NCX activity. In these experiments, UTP was used to evoke [Ca^2+^]_i_ transients.

### Immunofluorescent staining and semi-quantitative analysis

To investigate the effect of diabetes on the protein expression levels of SERCA, NCX and PMCA, diabetic and wild-type CECs were seeded in 24 well plates (6 wells per cell type) and allowed to spread. At 24 hours after seeding, cells were fixed in 2% paraformaldehyde. Immunofluorescent staining was performed by incubating the cells in rabbit anti-rat antibodies against SERCA, NCX and PMCA (abcam, MA) proteins, followed by incubation with Alexa Fluor 568-conjugated anti-rabbit secondary antibody (Invitrogen Corporation, CA). Cells were also stained with DAPI to identify cell nuclei (Invitrogen Corporation, CA). A total of 20 images for each protein were captured at 20X magnification using an Olympus IX81 microscope. The average fluorescent intensity of each image was quantified using MATLAB software (The Math Works, MA), and normalized by the intensity value of the corresponding negative control image (CECs incubated with only secondary antibody).

### Statistical analyses

The decay time of agonist-evoked Ca^2+^ transient was determined as the time in which [Ca^2+^]_i_ decayed from 80% to 20% of its peak amplitude (presented as t_80–20_). The results are expressed as mean ± SD. One way ANOVA (SPSS, IBM, IL) was used to determine the effect of diabetes on [Ca^2+^]_i_ decay kinetics and protein expression levels in CECs. A p<0.05 was considered to be statistically significant. All experiments were repeated at least 3 times; at least 70 diabetic/wild-type CECs were analyzed per treatment.
